# Quantification of progressive pulmonary fibrosis by visual scoring of HRCT images: recommendations from Italian chest radiology experts

**DOI:** 10.1007/s11547-025-01985-1

**Published:** 2025-04-07

**Authors:** Elisa Baratella, Andrea Borghesi, Lucio Calandriello, Giancarlo Cortese, Giovanni Della Casa, Chiara Giraudo, Emanuele Grassedonio, Anna Rita Larici, Stefano Palmucci, Chiara Romei, Ubaldo Romeo Plastina, Nicola Sverzellati

**Affiliations:** 1 Institute of Radiology, Department of Medical Surgical and Health Sciences, Cattinara Hospital, University of Trieste, Via Costantino Costantinides, 2, 34128 Trieste, Italy; 2 Department of Medical and Surgical Specialties, Radiological Sciences and Public Health, University of Brescia, ASST Spedali Civili of Brescia, Brescia, Italy; 3 Advanced Radiology Center - Department of Diagnostic Imaging, Oncological Radiotherapy and Hematology, A. Gemelli IRCCS University Polyclinic Foundation, Rome, Italy; 4 Hospital Maria Vittoria of Turin, Turin, Italy; 5 Azienda Ospedaliero-Universitaria of Modena, Modena, Italy; 6 Department of Cardiac-Thoracic and Vascular Sciences - DCTV, University of Padova, Padua, Italy; 7 Section of Radiology - Department of Biomedicine, Neurosciences and Advanced Diagnostics (BIND), University of Palermo, A.O.U.P. Giaccone, 90127 Palermo, Italy; 8 Section of Radiology - Department of Radiological and Hematological Sciences, Catholic University of the Sacred Heart, Rome, Italy; 9 Department of Medical, Surgical Sciences and Advanced Technologies, University of Catania – UOSD IPTRA, AOU Policlinico G. Rodolico-San Marco Di Catania, Catania, Italy; 10 Second Radiology Unit, Radiology Department, Pisa University Hospital, Pisa, Italy; 11 EcoRad Studio di Radiologia ed Ecografia, Reggio Calabria, Italy; 12 Radiological Sciences, Department of Medicine and Surgery, University of Parma, Parma, Italy

**Keywords:** Progressive pulmonary fibrosis, High-resolution computerized tomography, Fibrosis extent, Fibrosis progression, Consensus statements

## Abstract

Interstitial lung diseases (ILD) constitute a large and heterogeneous group of disorders affecting the lung parenchyma. While idiopathic pulmonary fibrosis (IPF), the most common type of ILD, is the prototype of progressive fibrosis, other forms, collectively termed “progressive pulmonary fibrosis” (PPF), can show a similar clinical course. Detecting chronic fibrosing ILD progression necessitates radiological evidence using high-resolution computed tomography (HRCT), which determines eligibility for treatment. However, assessing the extent of fibrosis and progression on HRCT images is difficult and lacks specific guidelines. Therefore, expert oversight and high-quality visual assessment/scoring of complex disease patterns is essential to monitor disease changes. Twelve Italian chest radiologists deliberated on the current state of quantifying lung fibrosis using existing literature to develop practice-oriented consensus statements to assist radiologists in visually assessing/scoring lung fibrosis on HRCT images in patients with PPF. The resulting statements cover three key areas: (1) technical requirements necessary for accurate HRCT image assessment; (2) an easy-to-use quantification protocol for routine clinical practice; and (3) a multiple specialist approach by combining radiological, clinical, and histopathological findings for the correct diagnosis, prompt detection of PPF, and timely start of antifibrotic treatment. In future, automated quantitative HRCT evaluation will lead to new clinical assessment tools.

**Graphical abstract:**

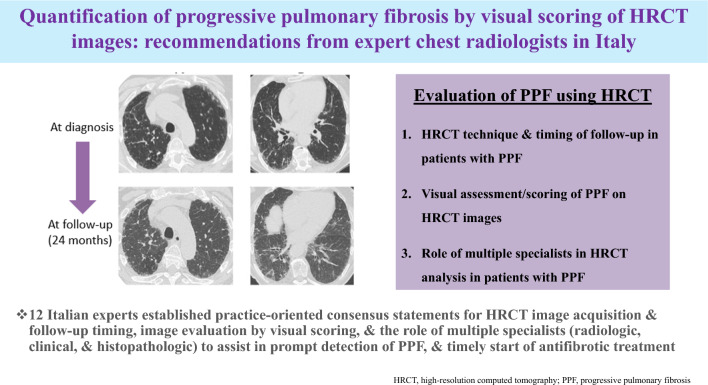

## Introduction

Interstitial lung diseases (ILD) constitute a large and heterogeneous group of disorders affecting the lung parenchyma [1, 2]. Idiopathic pulmonary fibrosis (IPF), a chronic, fibrosing interstitial pneumonia of unknown cause, is the most common type of ILD and the prototype of progressive fibrosis [3]. IPF shows histologic and radiologic features of usual interstitial pneumonia (UIP) [3, 4]; it affects older adults and is characterized by worsening of dyspnea, progressive decline in lung function, and a poor prognosis [3]. Besides IPF, other ILD can show the progression of lung fibrosis with a clinical course similar to IPF [2, 5, 6]. These ILD forms are generally termed progressive fibrosing ILD. In 2022, the updated guideline by the American Thoracic Society (ATS), European Respiratory Society (ERS), Japanese Respiratory Society (JRS), and the Latin American Thoracic Association (ALAT) adopted the term “progressive pulmonary fibrosis” (PPF) for this group of diseases [3].

Non-IPF diseases such as PPF is defined as at least two of three criteria (worsening symptoms, radiological progression, and physiological progression) that has occurred within the prior year with no alternative explanation in patients with an ILD other than IPF, and may progress over time [3]. Examples of PPF include fibrotic nonspecific interstitial pneumonia, chronic hypersensitivity pneumonitis, systemic sclerosis (SSc)-associated ILD, and connective tissue disease (CTD)-associated ILD [7].

Currently recommended medications for the treatment of IPF include the antifibrotic agents nintedanib and pirfenidone [8–11]. Based on the results from the INBUILD trial investigating the efficacy of nintedanib in ILD other than IPF, this antifibrotic agent has recently gained approval “for the treatment of other chronic fibrosing ILD with a progressive phenotype” [12], leading to a radical change in the management of ILD. This change is reflected in the efforts devoted by the 2022 ATS/ERS/JRS/ALAT guideline to define the progression of pulmonary fibrosis in ILD [3]. According to the guideline, progression is defined by the occurrence, over the past year, of at least two of the following events: worsening respiratory symptoms; physiological evidence of disease progression; and radiologic evidence of disease progression [3]. Indeed, progression of fibrosis on high-resolution computed tomography (HRCT) images was a stand-alone criterion for reduced transplant-free survival in a recent study that validated the proposed criteria for PPF [13]. The guideline also provides general radiologic criteria for both the visual determination and the quantitative assessment of fibrosis progression on HRCT images. This points to another important consequence of recent advances in the field, namely the increasingly central role of radiology, not only for diagnosis but also for longitudinal disease evaluation and the early detection of progression [4, 14, 15].

Due to the novelty of the extension of the nintedanib indication, several practical aspects are still poorly defined, including the optimal timing of antifibrotic treatment initiation in non-IPF fibrotic ILD and fibrosis assessment on HRCT images. For example, the eligibility criteria for nintedanib according to the Italian Medicines Agency (AIFA) require the presence of relevant lung fibrosis, defined as > 10% of fibrotic features on HRCT images in IPF [16]. This criterion may pose a challenge to clinicians, usually pulmonologists, who complete the AIFA eligibility form. In addition, radiologic examinations before patient referral to a specialist center are often performed by a general radiologist, who may lack expertise in chest HRCT and radiological features of fibrosis.

These uncertainties, along with the lack of specific guidelines for the evaluation of fibrosis on HRCT scans, prompted a group of chest radiologists from across Italy to convene and discuss the current state of lung fibrosis quantification; their ultimate goal was the development of practice-oriented statements to assist radiologists in the visual assessment/scoring of lung fibrosis on HRCT images. We report here the results of this effort, which is primarily directed to radiologists dealing with the analysis of HRCT images but may be of interest to multiple specialists involved in the management of patients with PPF at specialist centers.

## Methods

Twelve expert chest radiologists (the authors of this paper, with NS acting as the scientific coordinator) convened in two meetings (one virtual held in March 2022 and one in-person held in May 2022) to discuss current techniques of visual assessment of PPF on HRCT images and to address unresolved practical issues. The meetings were prompted by the need for simple, practice-oriented, and shared guidelines for analyzing HRCT images in routine clinical practice. The two preliminary meetings were followed by two virtual expert consensus meetings held in November 2022 and were attended by expert radiologists and a discussion facilitator. The role of the facilitator was to manage the discussion and help reach consensus using the MetaPlan^®^ technique.

During the first consensus meeting (November 4, 2022), three main areas were identified as relevant: (1) the optimization and standardization of HRCT image acquisition and timing of follow-up HRCT scans; (2) a shared protocol for the visual assessment/scoring of fibrosis on HRCT images; and (3) the role of different specialists in the analysis of HRCT images and in reporting fibrosis progression. Practical issues within each area that deserved to be addressed in detail were identified. The participants were then divided into three groups, one for each area of interest, and consensus statements were formulated addressing the unresolved issues in each area.

During the second consensus meeting (November 29, 2022), the statements were discussed further, finalized to the version presented here (Tables 1, 2 and 3), and voted to confirm the achievement of consensus. In detail, the tentative statements drafted by the members of the board were then discussed by the board as a whole. Following the discussion, in some cases, statements were reworded in order to improve clarity and accuracy; each statement was then submitted to the board, with the explicit request to each member to express their agreement or disagreement (each board member voted independently). Votes were then shown and evaluated, with the criterion of 2/3. (Each statement was approved in terms of consensus when at least 66.6% of voters expressed their agreement.)

**Table 1:** Statements about HRCT technique and timing of follow-up of patients with PPF

A. HRCT execution	
A1	The acquisition must be performed by volumetric HRCT at full inspiration
A2	Images must be reconstructed with a section ≤ 1.5 mm
A3	Images must be reconstructed with high spatial frequency algorithms and window settings suitable for lung parenchyma assessment
A4	HRCT is a non-contrast imaging technique
A5	The acquisition should be obtained in a supine position. However, if dorsal interstitial abnormalities are observed, then prone acquisition limited to lower lobes should be obtained
A6	A low-dose expiratory acquisition could/should be obtained to solve radiological doubts when inspiratory acquisition fails to clarify the clinical-radiologic situation (i.e., air trapping, tracheobronchomalacia)
A7	If the examination to be evaluated does not meet the requirements described in A1–A6, it may have to be repeated, depending on the clinical situation
A8	A follow-up HRCT should be performed 12 months after the previous radiological assessment, or earlier in case of clinical or functional impairment
A9	Images must be interpreted considering anamnestic data, clinical data, and previous radiologic examinations

*HRCT* high-resolution computed tomography *PPF* progressive pulmonary fibrosis

**Table 2:** Statements about the visual scoring of PPF on HRCT images

B. Visual evaluation of HRCT images	
B1	The coexistence of emphysema must be reported and quantified as < or ≥ 15%
B2	The extent of fibrosis abnormalities and emphysema must be considered globally in both lungs
B3	The baseline quantification of disease extent (expressed as a percentage) should be performed by visual scoring of five preselected regions: Region 1: the aortic arch; Region 2: 1 cm below the level of the carina; Region 3: the right pulmonary venous confluence; Region 4: the midpoint between Regions 3 and 5; and Region 5: 1 cm above the cupola of the right hemidiaphragm
B4	The extent of disease only refers to fibrotic abnormalities
B5	In the follow-up, visual scoring should consider the following features [4]: • Increased extent or severity of traction bronchiectasis • New ground-glass opacities with traction bronchiectasis • New fine reticulation • Increased extent or increased coarseness of reticular abnormality • New or increased honeycombing • Increased lobar volume loss Based on this assessment, the follow-up should be classified as improved, progressive, or stable
B6	Multiplanar reconstruction on sagittal and coronal planes must be evaluated to increase diagnostic confidence
B7	All features must be compared on corresponding anatomical levels

*HRCT* high-resolution computed tomography *PPF* progressive pulmonary fibrosis

**Table 3:** Statements about the roles of multiple specialists in HRCT analysis in patients with PPF

C. Roles of multiple specialists	
C1	The HRCT chest examination report of a patient with known or suspected fibrotic interstitial disease should contain a definition of the disease pattern, according to guidelines
C2	The quantification of the extent of fibrosis by percentage is required at baseline and follow-up examinations and should always be included in the structured report
C3	The quantification of the disease for therapeutic purposes involving multiple specialists should be based only on HRCT and not on ultrasound examinations
C4	Due to its potential therapeutic implications, the quantification of disease extent should be validated by an experienced thoracic radiologist and discussed among other specialists
C5	In case of confirmed fibrotic interstitial disease, the report should contain a recommendation for patient referral to an expert center with multiple specialists
C6	The radiological quantification should be integrated with clinical and functional data, with the involvement of other specialists

*HRCT* high-resolution computed tomography *PPF* progressive pulmonary fibrosis

## Results and discussion

The consensus statements covering the three areas of relevance (HRCT image acquisition, visual evaluation of fibrosis on HRCT images, and the role of multiple specialists involved in HRCT scan analysis) are reported in Tables 1, 2 and 3. General guidance on the diagnosis, follow-up, and management of patients with IPF and PPF are detailed in the latest ATS/ERS/JRS/ALAT guideline on IPF and PPF [3], the Fleischner Society White Paper on diagnostic criteria for IPF [4], and the Fleischner Society glossary of terms for thoracic imaging [17].

### HRCT image acquisition and timing of follow-up in patients with PPF

Adequate quality of the acquired image is crucial for accurately evaluating HRCT scans visually. Image quality should be assessed by expert chest radiologists. Standardized and shared protocols for image acquisition are required to ensure high quality and consistency, as well as to facilitate the comparisons of images between centers. Furthermore, if possible, the longitudinal assessment of fibrosis progression should be performed on serial images acquired in the same radiology facility with the same scanner.

In line with the literature and current national and international guidelines [4, 18, 19], the recommended acquisition protocol should meet the following requirements when diagnosing PPF (Table 1; Statements A1–A5): no use of contrast media (as lung parenchyma has by its nature a very high contrast); acquisition by volumetric scanning of the chest at full inspiration; in supine position; image reconstruction with thin sections (≤ 1.5 mm); high spatial frequency algorithm for lung parenchyma evaluation. Furthermore, to avoid movement artifacts, the acquisition time should be reduced by using the shortest rotation time (0.5 s) and the highest pitch [20, 21].

As the depth of pulmonary inspiration can influence lung attenuation, which may lead to variable images and misinterpretations, patients should be instructed on how to breathe before the examination and should receive further guidance during the examination [22]. At the first HRCT examination, expiratory acquisition is recommended if the inspiratory acquisition has not provided conclusive findings (Statement A6); expiratory acquisition can be used to identify air trapping, a feature observed in ILD-like hypersensitivity pneumonitis or CTD-ILD [4].

Acquisition in the prone position can be useful for investigating lung abnormalities observed in the dorsal areas of lower lobes on supine images and to distinguish between position-induced changes and interstitial changes (Statement A5) [4]. If the recommended technical requirements are not met and the image is of inadequate quality, according to the expert radiologist, a new HRCT examination may be necessary (Statement A7). The decision to repeat the HRCT examination also depends on patient clinical status. For example, repetition of HRCT is warranted in patients with clinical and functional findings of progressive fibrosis.

Follow-up HRCT scans are indicated when clinical or functional data suggest a worsening of fibrosis [3]. The optimal timing of follow-up HRCT scans to detect disease progression in patients with stable lung function is currently unknown. Evidence from studies in patients with SSc suggests that follow-up HRCT scans within 12–24 months from baseline examination should ensure early detection of progression and timely start of antifibrotic therapy [3]. Based on our experience, follow-up of patients with PPF within 12 months after the previous radiological assessment is advisable; in case of clinical and/or functional decline, the follow-up visit should be anticipated (Statement A8). The analysis and interpretation of HRCT images should consider patient medical history, clinical data, and previous radiological findings (Statement A9).

### Visual scoring of HRCT images in patients with PPF

The identification of traction bronchiectasis/bronchiolectasis and/or honeycombing on HRCT images is often sufficient for the diagnosis of lung fibrosis [3, 19]. IPF presents histologic and radiologic characteristics of UIP [3]. Typical radiologic UIP features visible on HRCT scans include honeycombing and traction bronchiectasis; the concomitant presence of ground-glass opacification and fine reticulation is also possible (the reader is referred to the 2018 ATS/ERS/JRS/ALAT guideline for the detailed description of these features and to the Fleischner Society glossary for proper terminology and definitions) [17, 20]. Furthermore, HRCT features have a prognostic value, with an increasing extent of the UIP pattern being associated with disease progression and mortality in IPF [3, 23–27].

In ILD, progression is characterized by more variable changes in HRCT images, including an evolution of the radiologic pattern along with an increase in the extent of fibrosis [3], which is measured at patient presentation (baseline) and has also been shown to have prognostic value. A study comparing software-based and visual evaluation of HRCT images in patients with IPF found that those with ≥ 10% of lung parenchyma affected by fibrosis at baseline had significantly worse outcomes than those with < 10%, after adjustment for relevant covariates and regardless of the quantification method used [28]. A greater extent of fibrotic HRCT changes is also predictive of mortality [29]. Moreover, honeycombing and traction bronchiectasis have been linked to a worse prognosis, not only in IPF but also in ILD associated with rheumatoid arthritis, SSc, chronic hypersensitivity pneumonitis, pulmonary sarcoidosis, and unclassifiable ILD [29].

The determination of radiologic fibrosis progression in clinical practice is currently performed based on the visual evaluation of HRCT images and relies on the estimated percentage of lung volume presenting fibrotic features (semi-quantitative visual scoring), which is then compared with findings from previous (baseline) HRCT scans [3]. The assessment of fibrosis progression is usually performed at specialist centers by a chest radiologist. Several methods have been proposed for semi-quantitative visual scoring of fibrosis on HRCT scans [23, 24, 30–33]. These methods typically divide axial chest HRCT images into several anatomically defined levels (usually, 5 or 6 levels) that are analyzed separately for the overall extent of disease in both lungs and for the contribution of the various features of UIP to abnormal lung, expressed as a percentage [24, 30].

**Fig. 1:**
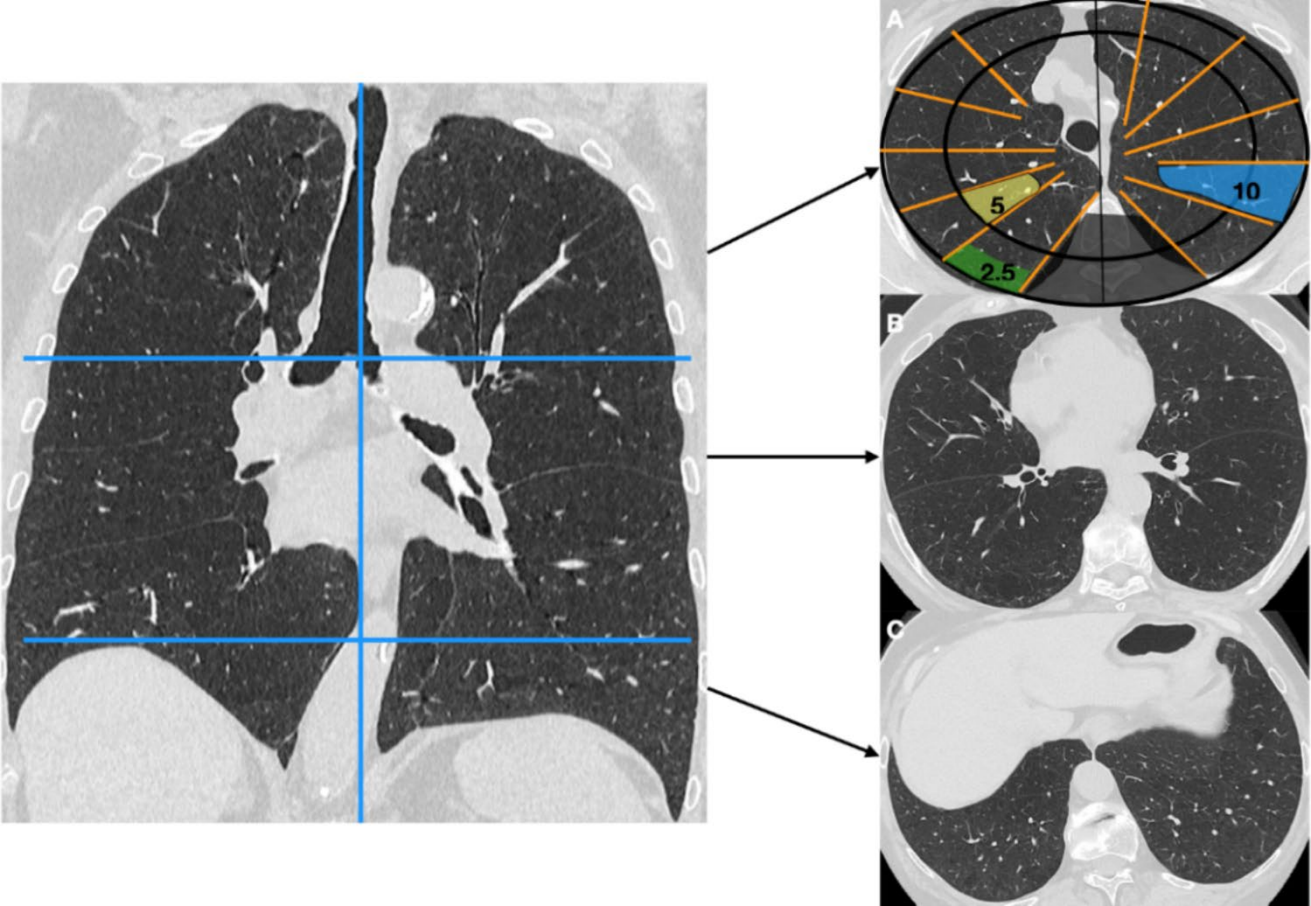
Visual score of pulmonary emphysema on computed tomography scans of three different levels of the lung (A = upper; B = middle; C = lower) are selected from high-resolution computed tomography then subdivided into smaller portions to determine the percentage of lung involvement

Emphysema is a common finding on scans of patients with ILD, especially in current or former smokers; the extent of emphysema > 15% of total lung volume must be reported because it is associated with comorbidities, a higher risk of complications (pulmonary hypertension and lung cancer), and disease progression [34]. Even though quantitative measures provide a reproducible and objective measurement of emphysema, it has been demonstrated that a visual score can identify not only the overall extent of emphysema but also additional morphological features, and can distinguish emphysema from small airways diseases and artifacts such as noise [35, 36]. According to Sánchez and colleagues, a visual score of pulmonary emphysema extent can be obtained by subdividing each lung into three zones (Fig. 1): upper (above the carina), lower (below the highest point of the right diaphragm), and middle (between the previous two) [37].

The extent of emphysema is also assessed to establish whether it spans ≥ or < 15% (Statements B1 and B2) [38]. The literature reporting on the use of these scores in clinical practice is limited, and no semi-quantitative visual scoring system has been shown to be superior to other visual methods.

**Fig. 2:**
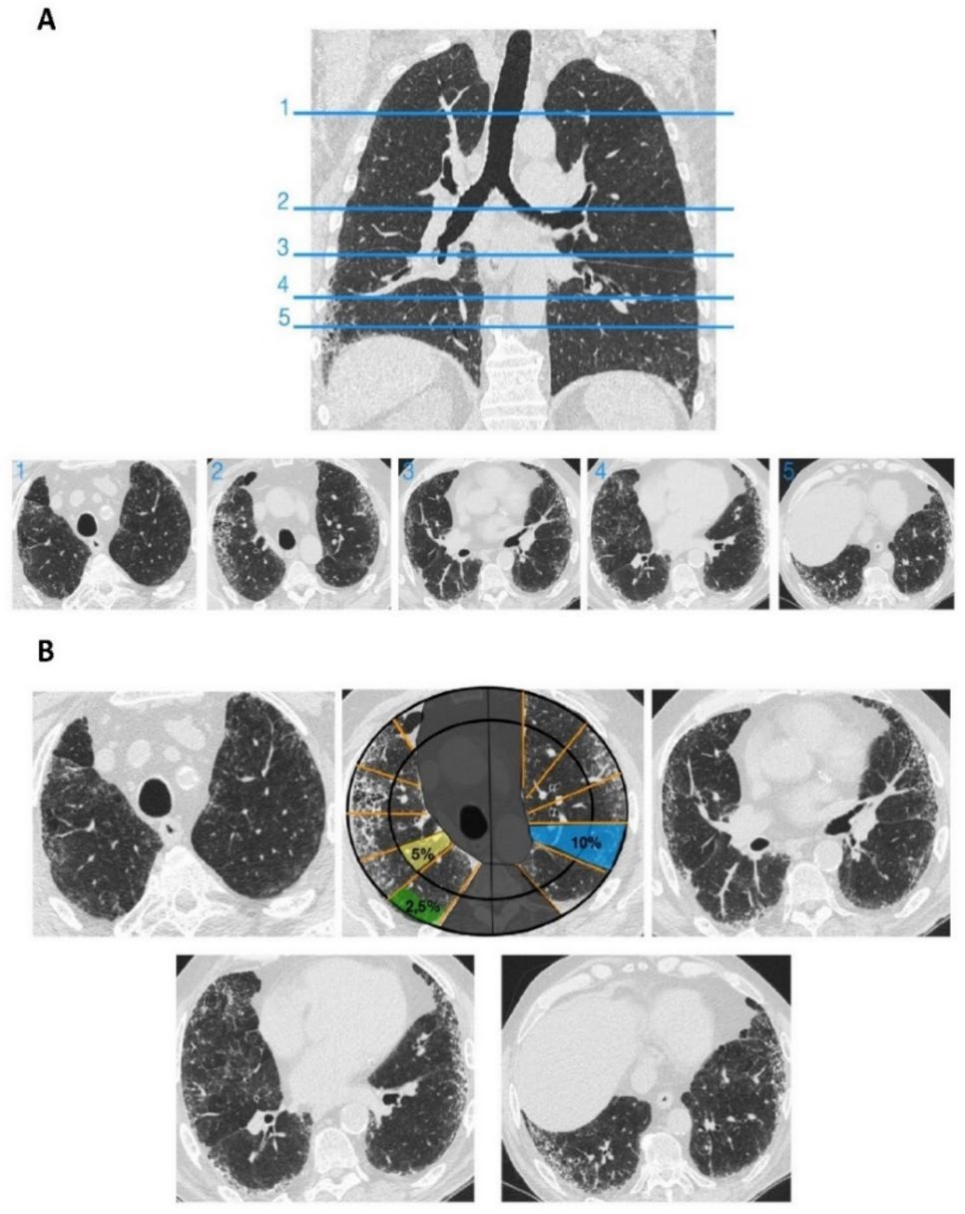
Proposal of a method for semi-quantitative assessment of the extent of fibrotic changes in patients with progressive pulmonary fibrosis. **A** High-resolution images at five preselected regions should be assessed: Region 1: the aortic arch; Region 2: 1 cm below the level of the carina; Region 3: the right pulmonary venous confluence; Region 4: the midpoint between 3 and 5; and Region 5: 1 cm above the cupola of the right hemidiaphragm. **B** Semi-quantification is performed for each of the five images considering each segment and subsegment as shown

The visual semi-quantitative protocol we propose in Table 2 for the assessment of HRCT images of patients with PPF uses five anatomical regions (Statement B3) as described by other authors [31, 32]. These levels adequately cover the upper, middle, and lower lung zones (Fig. 2A) [30].

**Fig. 3:**
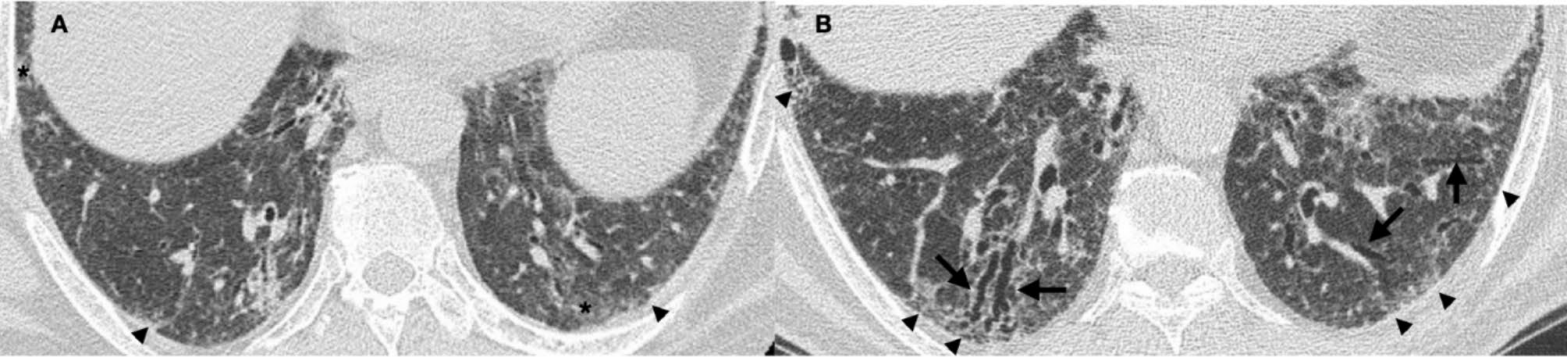
Traction bronchiectasis and disease progression in connective tissue disease-associated interstitial lung disease. **A** Baseline high-resolution computed tomography (HRCT) shows subtle ground-glass opacities (*) and fine reticulation (arrowheads) with no evidence of bronchiectasis. **B** Follow-up HRCT after 12 months demonstrates the disease progression with an increase of reticulation (arrowheads) and the appearance of newly developed bronchiectasis (arrows) in both lower lobes

The extent of fibrosis is determined by visually estimating the percentage of parenchymal involvement in each level and the percentages from the five levels are then averaged to obtain the overall extent of lung fibrosis (Statement B3) [24]. This can be done semi-quantitatively by dividing each lung into five segments corresponding to 10% of the lung parenchyma; a half of such a segment corresponds to 5% and one-quarter to 2.5%. (An example is given in Fig. 2B.) In addition, for each anatomical level, the presence and severity of traction bronchiectasis should be quantified as follows: grade 0 = none; grade 1 = mild; grade 2 = moderate; and grade 3 = severe [39, 40]. Taking into account the average of degree of airways dilatation in accordance with the Gestalt score [41], the extent of these alterations can be used to visually estimate the nearest 5% for each lung zone [33]. HRCT scans of traction bronchiectasis and disease progression in both lower lobes in CTD-ILD are shown on a baseline with no evidence of bronchiectasis (Fig. 3A), while a follow-up HRCT after 12 months demonstrated disease progression (increased reticulation and newly developed bronchiectasis (Fig. 3B).

**Fig. 4:**
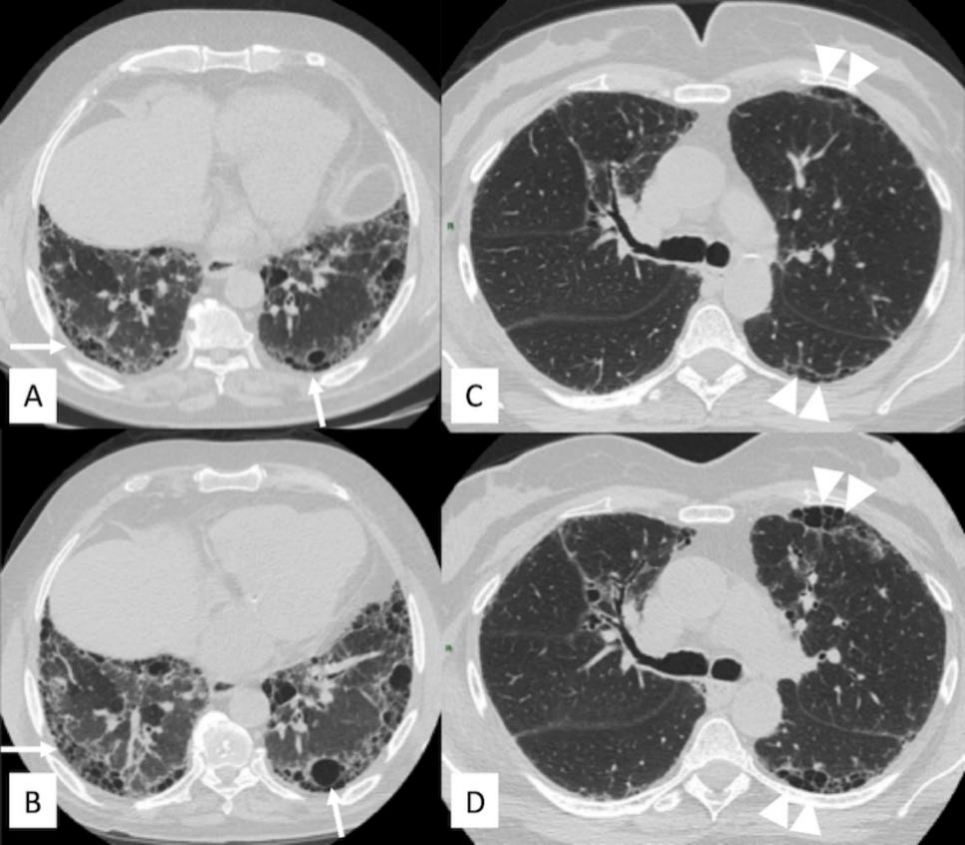
Progression of honeycombing in two different patients. Patient with fibrotic chronic hypersensitivity pneumonitis (CHP) with areas of honeycombing (white arrows) in the lower lobes at **A** baseline and **B** after 16 months of follow-up, where an increase in extent and severity of honeycombing are depicted on the periphery of both lower lobes. Patient with fibrotic connective tissue disease (CTD) with honeycomb spaces (white arrowheads) in the anterolateral region of the left upper lobe and in the posterosuperior region of the left lower lobe at **C** baseline and **D** after 12 months of follow-up: honeycomb is more represented in the follow-up high-resolution computed tomography examination

**Fig. 5:**
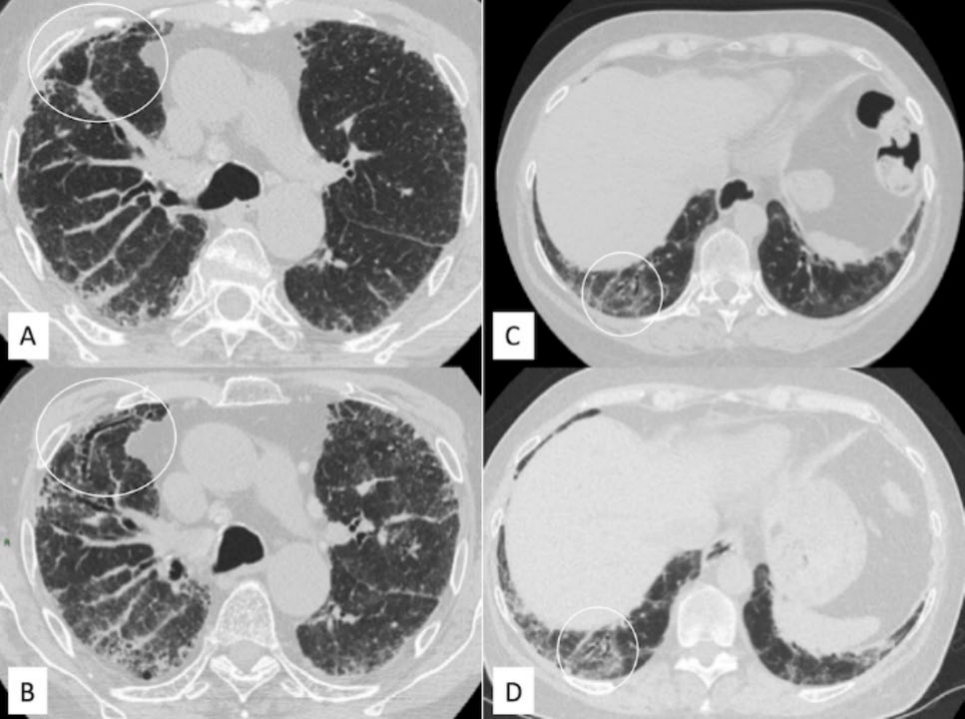
Traction bronchiectasis and stable bronchiectasis. **A**, **B** Traction bronchiectasis (white circles), which are increased in extension and caliper in the follow-up examination. **C**, **D** Stable bronchiectasis of the right lower lobe between high-resolution computed tomography examinations, even if progression is clearly demonstrated by increased representation of ground-glass opacification close to bronchiectasis

**Fig. 6:**
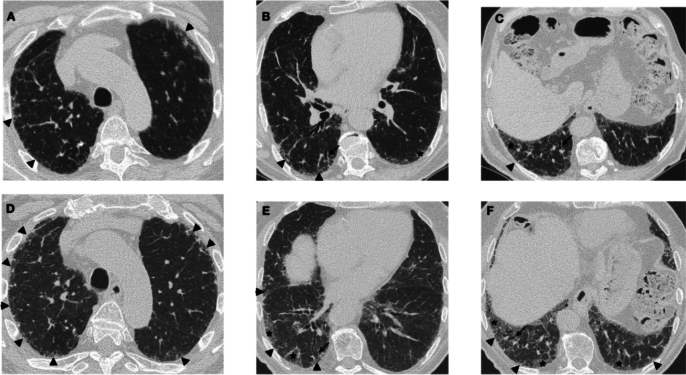
Disease trajectories in a patient with systemic sclerosis-associated interstitial lung disease and a progressive fibrotic phenotype. **A**–**C**. Images at diagnosis: Axial high-resolution computed tomography (HRCT) images show diffuse subpleural reticulation (arrowheads), subtle ground-glass opacities (*) and traction bronchiolectasis (arrows). **D**–**F**. Images at follow-up: HRCT demonstrates disease progression after 24 months due to the increased extent of ground-glass opacities (*) and reticulation (arrowheads), in association with an increased extent and severity of traction bronchiectasis (arrows)

It should be noted that the extent of disease only refers to fibrotic abnormalities to be assessed globally (i.e., both lungs; Statements B2 and B4). The areas to be considered are those affected by (i) honeycombing (Fig. 4), (ii) traction bronchiectasis (Fig. 5), (iii) reticulations (Fig. 3), and (iv) ground-glass opacities (Fig. 6), when associated with fibrosis/architectural distortion. All features must be compared on corresponding anatomical levels (Statement B5). Multiplanar reconstruction on sagittal and coronal planes must be evaluated to increase diagnostic confidence (Statement B6).

**Fig. 7:**
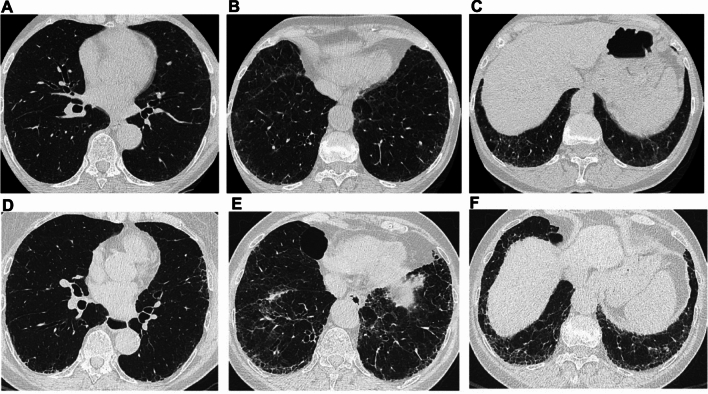
Disease trajectories in patients with systemic sclerosis-associated interstitial lung disease and a progressive fibrotic phenotype. **A**–**C**. Images at diagnosis. **D**–**F**. Images at follow-up, which demonstrate disease progression after 24 months

**Fig. 8:**
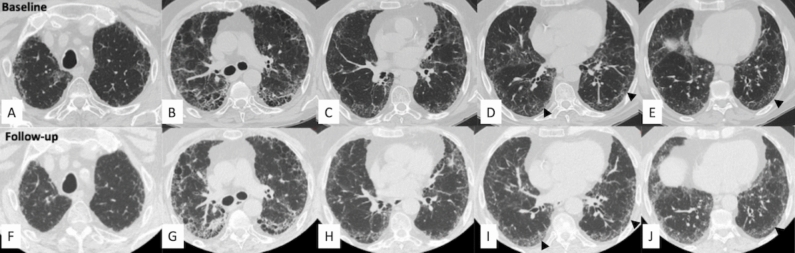
Disease progression, assessed through a side-by-side comparison of high-resolution computed tomography (HRCT) examinations in a patient with PPF secondary to myositis. Baseline HRCT scans (**A**–**E**) and follow-up HRCT scans (**F**–**J**) were evaluated considering imaging features of five thoracic levels: ground-glass opacifications and reticulations are slightly more represented in the basal regions (black arrowheads). In the assessment of fibrotic disease progression, a side-by-side comparison is strongly recommended in order to reduce variability and to increase reproducibility among radiologists and clinicians

The fibrotic features to be considered in the follow-up of patients with PPF (Statement B7) should include increased traction bronchiectasis, new ground-glass opacities with traction bronchiectasis, new fine reticulation, increased extent or increased coarseness of reticular abnormalities, new or increased honeycombing, and increased lobar volume loss, as pointed out in the 2022 ATS/ERS/JRS/ALAT guideline [3]. Based on the results of this assessment, PPF would be classified as improved, stable, or progressive. At follow-up, determining disease extent is not as strictly required as at baseline because it may be sufficient to report whether the radiologic pattern has remained unchanged or worsened from baseline (i.e., increased extent of previous features and/or occurrence of new features). Notably, HRCT examination alone is usually not sufficient to establish disease progression with confidence and should be supported by clinical and functional evaluations. Notably, some HRCT abnormalities may improve or even resolve over time. This is usually observed for either ground-glass opacities or consolidation in fibrotic disorders associated with an inflammatory component (e.g., CTD or hypersensitivity pneumonitis) [42, 43]. Examples of disease trajectories in SSc-ILD and PPF secondary to myositis are shown in Figs. 7 and 8, respectively.

Over the past decade, several computer-assisted methods for the quantitative assessment of HRCT images have been developed, with promising results in terms of sensitivity, reliability, and consistency [28, 44–49]. However, their use in clinical practice is still limited and there is currently no consensus on which method should be used. Thus far, two automated systems for the quantification of lung fibrosis on HRCT images have received the CE mark from regulatory agencies in Europe, namely the CALIPER, commercialized under the name of IMBIO, and Coreline. However, both systems are often unavailable in everyday clinical practice.

Furthermore, there are other aspects concerning these systems that still need validation, especially in terms of reproducibility since lung texture analysis may be affected by patient characteristics (i.e., lung volume, breath hold duration during CT scan acquisition, change in smoking status) or related to the scanner (i.e., calibration, radiation dose, acquisition and reconstruction protocols) [49–51].

### Role of multiple specialists in the visual evaluation and medical reporting of PPF

Within specialist centers, the radiologist often collaborates with other specialists, such as the pulmonologist and the pathologist [4]. Evidence from studies evaluating the impact of multiple specialists on the diagnosis and management of ILD has shown that discussion between specialists is associated with elevated rates of diagnosis changes and subsequent alterations of management (about 40% for both) [52]. Collaboration between radiologists, pulmonologists, and pathologists involved in the care of patients with ILD has been shown to enhance interobserver agreement, increase diagnostic confidence, and facilitate accurate diagnosis, particularly in cases with uncertain initial diagnoses [53–55]. A multiple specialist approach is particularly useful in PPF and when disease is unclassifiable according to standard criteria [52, 54].

Radiologists are responsible for writing a report of the HRCT examination, which should contain information on key signs and ancillary features of fibrosis to provide a comprehensive description of the ILD pattern [20, 56]. The report should use the terminology defined by current guidelines for the description of radiological features of UIP (Table 3; Statement C1) [3, 17]. As recommended by Italian guidelines for written chest radiology structured reports, the extent of lung fibrosis should be systematically reported as a percentage of the total lung volume at baseline (Statement C2) [57]. The use of structured templates have been encouraged by many scientific societies and previous studies, which emphasizes that structured templates should be used as a checklist or diagnostic algorithm [58]. Specific items for quantification of the extent of fibrotic disease should be embedded in templates to provide an exhaustive and accurate response to clinicians [58]. Until standardized reporting templates are developed, it is strongly recommended to reserve the use of structured templates in daily clinical practice to an experienced chest radiologist.

Importantly, the visual quantification of disease extent must be performed exclusively on HRCT scans and not on ultrasound examinations (Statement C3). Due to the therapeutic relevance recently acquired by the estimated fibrosis extent, which is one of the criteria required by the Italian medication agency for selecting patients with ILD eligible to antifibrotic treatment with nintedanib, the radiology report should be reviewed and validated by a chest radiologist if the HRCT examination has been performed by a general radiologist in a non-referral center (Statements C2 and C4) [14, 15, 17]. In particular, the expert chest radiologist is required to confirm key signs and ancillary features of fibrosis and the estimated percentage of disease extension, as well as to evaluate the technical quality of the examination as detailed in the section mentioned above on HRCT image acquisition and timing of follow-up.

For patients undergoing their first HRCT examination at a non-referral center, and in case of confirmed fibrotic interstitial disease, the radiology report should express a recommendation for patient referral to an expert center (Statement C5). A multiple specialist approach is essential for completing and confirming the radiologic examination, with clinical and functional data provided by the pathologist and the pulmonologist (Statement C6). At the same time, the radiologist’s contribution is crucial for the pulmonologist (responsible for filling in the form requested by the AIFA) to confirm both patient eligibility for nintedanib treatment, as well as radiologic data. Even more importantly, the expert radiologist may play a key role in the early detection of progressive fibrosis [14, 15, 17].

## Conclusion

Recent advances have highlighted the importance of the radiologic assessment of PPF. While acknowledging the potential of HRCT imaging in terms of precision and consistency, we believe that the role of expert chest radiologists remains essential in ensuring high-quality visual assessment of complex disease patterns and in the monitoring of disease changes. The consensus statements presented here are intended to help radiologists estimate the extent of fibrosis on HRCT images in patients with PPF. The statements focus on three important components of the radiologic examination, namely the technical requirements necessary for accurate HRCT image assessment, an easy-to-use quantification protocol for routine clinical practice, and a multiple specialist approach that combines radiologic, clinical, and histopathologic findings for a correct diagnosis, prompt detection of progressive fibrosis, and timely start of antifibrotic treatment. The rapid development of automated, quantitative HRCT image evaluation may soon lead to the introduction of new assessment tools in clinical practice.

## Data Availability

Data sharing is not applicable to this article as no datasets were generated or analyzed during the current study.

## Declarations

### Authors declarations

The authors meet the criteria for authorship as recommended by the International Committee of Medical Journal Editors (ICMJE). They did not receive any payment related to the development of the subject manuscript.
