# Correction: Quantification of progressive pulmonary fibrosis by visual scoring of HRCT images: recommendations from Italian chest radiology experts

**DOI:** 10.1007/s11547-025-02036-5

**Published:** 2025-06-27

**Authors:** Elisa Baratella, Andrea Borghesi, Lucio Calandriello, Giancarlo Cortese, Giovanni Della Casa, Chiara Giraudo, Emanuele Grassedonio, Anna Rita Larici, Stefano Palmucci, Chiara Romei, Ubaldo Romeo Plastina, Nicola Sverzellati

**Affiliations:** 1 Institute of Radiology, Department of Medical Surgical and Health Sciences, Cattinara Hospital, University of Trieste, Via Costantino Costantinides, 2, 34128 Trieste, Italy; 2 Department of Medical and Surgical Specialties, Radiological Sciences and Public Health, University of Brescia, ASST Spedali Civili of Brescia, Brescia, Italy; 3 Advanced Radiology Center - Department of Diagnostic Imaging, Oncological Radiotherapy and Hematology - A. Gemelli IRCCS University Polyclinic Foundation, Rome, Italy; 4 Hospital Maria Vittoria of Turin, Turin, Italy; 5 Azienda Ospedaliero-Universitaria of Modena, Modena, Italy; 6 Department of Cardiac-Thoracic and Vascular Sciences - DCTV, University of Padova, Padua, Italy; 7 Section of Radiology - Department of Biomedicine, Neurosciences and Advanced Diagnostics (BIND), University of Palermo, A.O.U.P. Giaccone, 90127 Palermo, Italy; 8 Section of Radiology - Department of Radiological and Hematological Sciences, Catholic University of the Sacred Heart, Rome, Italy; 9 Department of Medical, Surgical Sciences and Advanced Technologies, University of Catania – UOSD IPTRA, AOU Policlinico G. Rodolico-San Marco Di Catania, Catania, Italy; 10 Second Radiology Unit, Radiology Department, Pisa University Hospital, Pisa, Italy; 11 EcoRad Studio Di Radiologia Ed Ecografia, Reggio Calabria, Italy; 12 Radiological Sciences, Department of Medicine and Surgery, University of Parma, Parma, Italy

**Correction to: La radiologia medica** 10.1007/s11547-025-01985-1

In the published version, author would like to the add the “Authors Declarations” section in the backmatter of the article as given below:

Authors Declarations: The authors meet the criteria for authorship as recommended by the International Committee of Medical Journal Editors (ICMJE). They did not receive any payment related to the development of the subject manuscript.

